# Implementing new advanced airway management standards in the Hungarian physician staffed Helicopter Emergency Medical Service

**DOI:** 10.1186/s13049-014-0081-z

**Published:** 2015-01-09

**Authors:** Akos Soti, Peter Temesvari, Laszlo Hetzman, Attila Eross, Andras Petroczy

**Affiliations:** Hungarian Air Ambulance Nonprofit Ltd., Legimentok u. 8, Budaors, H-2040 Hungary; National Ambulance Service, Robert K. krt 77, Budapest, H-1134 Hungary

**Keywords:** Prehospital, Rapid sequence intubation, Advanced airway, Success rate, Helicopter emergency medical service

## Abstract

In 2011 the Hungarian Air Ambulance Nonprofit Limited Company introduced a new Rapid Sequence Intubation standard operating procedure using a template from London’s Air Ambulance. This replaced a previous ad-hoc and unsafe prehospital advanced airway management practice. It was hoped that this would increase clinical standards including internationally comparable results. All Rapid Sequence Intubations performed by the units of the Hungarian Air Ambulance under the new procedure between June 2011 and November 2013 were reviewed in a retrospective database analysis. During this period the air ambulance units completed 4880 missions with 433 intubations performed according to the new procedure. The rate of intubations that were successful on first attempt was 95.4% (413), while intubation was successful overall in 99.1% (429) of the cases; there was no failed airway. 90 complications were noted with 73 (16.9%) patients. Average on scene time was 49 minutes (ranging between: 15–110 minutes). This data shows that it is possible to effectively change a system that was in place for decades by implementing a new robust system that is based on a good template.

## Background

The Hungarian Air Ambulance Nonprofit Ltd. is the only Helicopter Emergency Medical Service (HEMS) provider in Hungary operating from 7 air bases with 100% physician manned helicopters in daylight hours. Prehospital endotracheal intubation has been routine in the last few decades in Hungary, but until recently, no effort was made to standardize indications or procedures for drug assisted intubation. Muscle relaxant drugs were never used (not even by anaesthetists) in prehospital care. This practice has never been thoroughly audited nor any data published on it. In 2010 the results of a local audit strengthened the suspicion that the previously described practice didn’t meet internationally published standards. Although the overall success rate of intubation was 100% in these cases, first attempt success rate was well below optimal and a high complication rate suggested the system might be unsafe. (n: 38, success: 100%, success on first attempt: 68%, complications: 39%) [Hungarian Air Ambulance, non-published data].

The need for prehospital advanced airway management (especially endotracheal intubation) is still a debated topic [1,2]. Prehospital endotracheal intubation only improves outcome if performed in a governance system focused on patient safety [3]. In 2011 a decision was made to change the ad hoc and unsafe practice and to implement a new RSI Standard Operating Procedure (SOP) for the HEMS units. The aim was to achieve high clinical standards and internationally comparable results.

## Standard operating procedure and clinical governance

**Figure 1:**
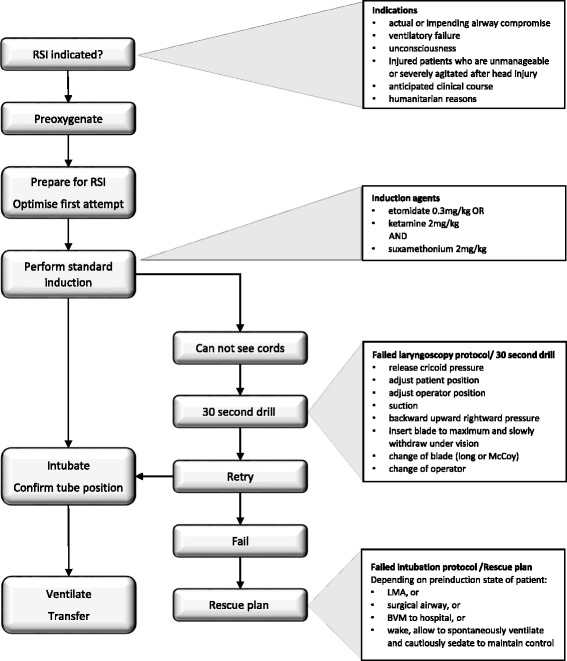
**RSI algorithm.**

Authors used the SOP of London’s Air Ambulance with their help and consent as a template [4]. The reasons behind this decision were that this SOP was highly successful with the London Service, the authors had first-hand experience with it and it is a part of a robust Clinical Governance framework [5,6]. One of the key features of this SOP is to optimize the first attempt at laryngoscopy while providing backup plans for failed laryngoscopy and for failed intubation [Figure 1].

Before intubation, the prehospital team goes through a challenge-response checklist. Application of cricoid pressure was optional, with a low threshold to release if it obstructed the operator’s view [7,8].

Implementing the SOP came with building up the elements of the earlier non-existent clinical governance as well [6,9]. There was a need for some drugs and extra equipment and also to rearrange the equipment carried by teams. All HEMS staff took part in simulation training over two days with a qualifying exam on completion. This required first training the instructors by those with recent UK (United Kingdom) HEMS experience.

Continuous refresher courses were introduced to establish currency after implementing the system. All HEMS staff and all RSI cases were audited on a regular basis. We have implemented an on call consultant system, with consultants available via phone during service hours – we expected clinicians to make a short phone consultation before every procedure.

## Method

We have completed a retrospective database audit on all bases involved from June 2011 till November 2013, summing 30 months of data. All patients intubated by HEMS using RSI were included. Cardiac arrests and patients not intubated by HEMS were excluded. We looked at the number of attempted laryngoscopies, failed laryngoscopy protocol elements used, and also documented any complications in different patient sub-groups. An intubation attempt was defined as attempted laryngoscopy with the intent to intubate. Blood pressure increase or decrease was recorded as a complication when the change after induction was more than 20% compared to the pre-induction value. Desaturation was recorded as a drop of saturation after induction to below 90%. Aspiration was identified as any tracheal soiling of saliva, gastric contents or blood. We have identified bleeding as any bleeding caused by laryngoscopy. Bradycardia was documented when heart rate went below 50/min. Complications had to be procedure related (not present before induction). We also documented on scene times. For data collection we have used the patient documentation software “Esetlap 2006” by Tamas Gaspar. For further analysis, Microsoft Office Excel^©^ 2013 was used.

## Results

In the observed period HEMS units completed 4880 missions, and performed 433 intubations following the new RSI SOP. Mean patient age was 46 ± 22 year (4 month – 97), with the sex ratio of 72% male and 28% female. Indications are shown in Table 1.

**Table 1:** **Indications for RSI**

**Indications for RSI**	**n**	**%**
Actual or impending airway compromise	42	9.7
Ventilatory failure	56	12.9
Unconsciousness	271	62.6
Injured patients who are unmanageable or severely agitated after head injury	51	11.8
Anticipated clinical course	13	3.0
Humanitarian reasons	0	0.0

Success of first attempt at laryngoscopy was recorded in 413 (95.4%) cases. A second laryngoscopy was successful in 15 cases (3.5%), and in one case (0.2%) a third attempt was needed to pass the tube. Altogether out of the 433 patients we have managed to successfully intubate 429 (99.1%).

In the remaining 4 cases (0.9%) the intubation was unsuccessful. In 2 cases the airway was managed with a LMA, in the other 2 cases with surgical airway. Failed airway was not documented [Table 2].

**Table 2:** **Sub-group analysis of laryngoscopy attempts, complications and on scene time**

		**Summary**		**Trauma**										**Medical**													
		**n: 433 (100%)**		**All trauma**		**Isolated head injury**		**Burns**		**Hanging**		**Other**		**All medical**		**Stroke**		**Intoxication**		**Status epilepticus**		**Ventilatory failure**		**Post - ROSC**		**Other**	
				**65.1%**		**19.1%**		**6.7%**		**2.1%**		**37.2%**		**34.9%**		**8.3%**		**7.2%**		**5.1%**		**4.2%**		**3.2%**		**6.9%**	
		**n**	**%**	**n**	**%**	**n**	**%**	**n**	**%**	**n**	**%**	**n**	**%**	**n**	**%**	**n**	**%**	**n**	**%**	**n**	**%**	**n**	**%**	**n**	**%**	**n**	**%**
**Attempts at laryngoscopy**	1th	**413**	**95.4**	**269**	**95.4**	80	96.4	27	93.1	9	100	153	95.0	**144**	**95.4**	36	100	28	90.3	21	95.5	18	100	12	85.7	29	96.7
	2th	**15**	**3.5**	**10**	**3.5**	3	3.6	1	3.4			6	3.7	**5**	**3.3**			2	6.5	1	4.5			1	7.1	1	3.3
	3th	**1**	**0.2**	**1**	**0.4**							1	0.6														
	Failed	**4**	**0.9**	**2**	**0.7**			1	3.4			1	0.6	**2**	**1.3**			1	3.2					1	7.1		
**Complications**	Hypotension	**35**	**8.1**	**23**	**8.2**	4	4.8	3	10.3	2	22.2	14	8.7	**12**	**7.9**			4	12.9	3	13.6	4	22.2	1	7.1		
	Desaturation	**35**	**8.1**	**19**	**6.7**	4	4.8	1	3.4	1	11.1	13	8.1	**16**	**10.6**	4	11.1			3	13.6	2	11.1	4	28.6	3	10.0
	Aspiration	**5**	**1.2**	**3**	**1.1**	1	1.2	1	3.4			1	0.6	**2**	**1.3**									2	14.3		
	Bleeding	**5**	**1.2**	**2**	**0.7**	1	1.2					1	0.6	**3**	**2.0**			1	3.2					2	14.3		
	Misplacement	**3**	**0.7**	**3**	**1.1**	2	2.4					1	0.6														
	Hypertension	**3**	**0.7**	**1**	**0.4**	1	1.2							**2**	**1.3**	1	2.8									1	3.3
	Dental injury	**2**	**0.5**	**1**	**0.4**							1	0.6	**1**	**0.7**									1	7.1		
	Bradycardia	**2**	**0.5**	**1**	**0.4**							1	0.6	**1**	**0.7**					1	4.5						
**On scene time (hh:mm)**	Mean	**0:49**		**0:47**		**0:46**		**0:49**		**0:44**		**0:53**		**0:53**		**0:50**		**0:51**		**0:59**		**0:54**		**0:45**		**0:59**	
	Standard deviation	0:14		0:12		0:13		0:11		0:11		0:12		0:16		0:16		0:13		0:18		0:18		0:12		0:18	
	Minimum	0:15		0:20		0:20		0:25		0:31		0:21		0:15		0:20		0:20		0:32		0:30		0:15		0:35	
	Maximum	1:50		1:32		1:32		1:17		1:01		1:31		1:50		1:21		1:23		1:50		1:23		1:04		1:49	

The 30 second drill (failed laryngoscopy protocol) was used in 93 patients (21.5%) in different combinations adding up to 154 elements used altogether [Table 3]. Cricoid pressure was applied in only 13 (3%) cases. Out of these cases, it had to be released in 7 incidents (53.8% of the cases where it was applied).

**Table 3:** **Elements of failed laryngoscopy protocol**

**Elements of failed laryngoscopy protocol (30 second drill)**	**n**	**%**
Release cricoid pressure	7	1.6
Adjust patient position	9	2.1
Adjust operator position	9	2.1
Suction	55	12.7
Backward upward rightward pressure (BURP)	39	9.0
Insert blade to maximum and slowly withdraw under vision	22	5.1
Change of blade	9	2.1
Change of operator	4	0.9

There were 90 complications in 73 patients. The mean on scene time was 49 ± 14 (15–110 minutes). Sub-group analyses are shown in Table 2.

## Discussion

Hungarian prehospital advanced airway management was previously based on ad hoc practice and individual decision making. With the implementation of the new robust RSI SOP we have achieved our aim to implement high clinical standards and allow our findings to be compared with those published internationally [5,10-17]. Patient safety has improved, and not only by those treated but also by positive feedback from crews and hospital staff.

We are absolutely convinced that establishing a clinical governance system in parallel with the implementation of the new SOP was key in achieving our results. Our collected data suggests that it is possible to effectively change a flawed system that was in place for decades with a lot of commitment and implementing a new robust system that is based on a good template.

## Abbreviations

BURP: Backward upward rightward pressure
BVM: Bag-valve-mask
ERC: European Resuscitation Council
GCS: Glasgow Coma Scale
HEMS: Helicopter Emergency Medical Service
LMA: Laryngeal Mask Airway
RSI: Rapid Sequence Intubation
SOP: Standard Operating Procedure
UK: United Kingdom
